# Minimally-invasive cardiac surgery: a bibliometric analysis of impact and force to identify key and facilitating advanced training

**DOI:** 10.1186/s13019-022-01988-3

**Published:** 2022-09-16

**Authors:** Rickesh Bharat Karsan, Rhian Allen, Arfon Powell, Gwyn William Beattie

**Affiliations:** 1 Department of Cardiothoracic Surgery, Royal Victoria Hospital, 274 Grosvenor Road, Belfast, BT12 6BA UK; 2 Department of Cardiothoracic Surgery, University Hospital Wales, Cardiff, CF14 4XW UK; 3 Division of Cancer and Genetics, University Hospital of Wales, Cardiff, CF14 4XW UK; 4 Department of Surgery, University Hospital of Wales, Cardiff, CF14 4XW UK

**Keywords:** Minimally-invasive surgery, Cardiac surgery, Bibliometric analysis, Training

## Abstract

**Background:**

The number of citations an article receives is a marker of its scientific influence within a particular specialty. This bibliometric analysis intended to recognise the top 100 cited articles in minimally-invasive cardiac surgery, to determine the fundamental subject areas that have borne considerable influence upon clinical practice and academic knowledge whilst also considering bibliometric scope. This is increasingly relevant in a continually advancing specialty and one where minimally-invasive cardiac procedures have the potential for huge benefits to patient outcomes.

**Methods:**

The Web of Science (Clarivate Analytics) data citation index database was searched with the following terms: [Minimal* AND Invasive* AND Card* AND Surg*]. Results were limited to full text English language manuscripts and ranked by citation number. Further analysis of the top 100 cited articles was carried out according to subject, author, publication year, journal, institution and country of origin.

**Results:**

A total of 4716 eligible manuscripts were retrieved. Of the top 100 papers, the median (range) citation number was 101 (51–414). The most cited paper by Lichtenstein et al. (Circulation 114(6):591–596, 2006) published in Circulation with 414 citations focused on transapical transcatheter aortic valve implantation as a viable alternative to aortic valve replacement with cardiopulmonary bypass in selected patients with aortic stenosis. The Annals of Thoracic Surgery published the most papers and received the most citations (n = 35; 3036 citations). The United States of America had the most publications and citations (n = 52; 5303 citations), followed by Germany (n = 27; 2598 citations). Harvard Medical School, Boston, Massachusetts, published the most papers of all institutions. Minimally-invasive cardiac surgery pertaining to valve surgery (n = 42) and coronary artery bypass surgery (n = 30) were the two most frequent topics by a large margin.

**Conclusions:**

This work establishes a comprehensive and informative analysis of the most influential publications in minimally-invasive cardiac surgery and outlines what constitutes a citable article. Undertaking a quantitative evaluation of the top 100 papers aids in recognising the contributions of key authors and institutions as well as guiding future efforts in this field to continually improve the quality of care offered to complex cardiac patients.

## Background

The advent of interventional cardiology has preceded changes of patient demographics in Cardiac Surgery, with prospective patients being older and arguably much more complex [1]. Minimally-invasive techniques have started to become more prominent as cases become more complex. As of the mid-1990s there are been development of techniques to involve mini-sternotomy and mini-thoracotomy approaches, this however currently appears to be more surgeon and centre specific due to high complexity of such procedures [2, 3].

In accordance with the American Heart Association definition, alternative approaches not involving the traditional full sternotomy belong to the class of minimally-invasive cardiac surgery (MICS) [4]. With the use of such techniques however, it is hoped that there are improved patient outcomes and reduced risks to patients by way of shorter hospital admissions, earlier return to normal daily activity and lower post-operative infection rates as opposed to full sternotomy approaches. The evidence however is currently limited with current evidence showing post-operative outcome comparable to traditional techniques [5]. Research into minimally-invasive cardiac surgery is ongoing with significant studies such as the UK Mini-mitral trial (Minimally-invasive thoracoscopically-guided right minithoracotomy versus conventional sternotomy for mitral valve repair) and Manubrium-limited ministernotomy versus conventional sternotomy for aortic valve replacement (MAVRIC) trial [6].

With continued research and development, MICS is set to become highly prevalent in Cardiothoracic Surgery training and identifying key areas of research would help to guide training and research in the future.

Citations are gathered when an article is referenced by another peer-reviewed paper.

The number of citations a paper receives is considered to reflect the impact a paper has in the scientific community, as such those bodies of work with the greatest number of citations are considered to have the greatest impact. These articles will therefore have the greatest bearing on current surgical opinion and are likely to influence surgical training [7]. Ellul et al. [8] have previously used such an analysis to determine research themes that are most influential in understanding emergency abdominal surgery pathology and management to ultimately guide future research. Within general cardiac surgery, it has been suggested that despite some flaws, bibliometric analysis has inherent merits to guiding future research and potentially influencing training [9].

Currently no bibliometric analysis has been undertaken to determine the significant manuscripts in MICS. The aim of this study was to determine the studies most influential in the current development of MICS based on bibliometric scope. Identify the core topics and themes researched in this emergent class of cardiac surgery, leading to better understanding training needs in Cardiothoracic Surgery.

## Materials and methods

The Web of Science (Clarivate Analytics) data citation index database was searched with the following terms: [Minimal* AND Invasive* AND Card* AND Surg*]. Results were limited to full text English language manuscripts and ranked by citation number.

Results were limited to full text English Language papers account for the whole time period encompassed. A team of cardiac surgeons and trainees (GB, RK, RA) conducted a final analysis of the Web of Science results to identify the top 100 cited papers that were found to be relevant to MICS. Further regression analysis was conducted of the 100 papers with the most citations. Analyses were performed by author, subject, year of publication, journal, journal impact factor, institution and originating country.

A citation rate variable was formulated for each identified paper, by dividing the total number of citations by the number of years since publication. Articles with the same number of citations were ranked according to citation rate. Further regression analysis was performed to ascertain a potential relationship between journal impact factor and citation using SPSS v23.0. Results were considered significant if *P* ≤ 0.05.

## Results

A total of 4716 full articles were retrieved via the Web of Science, all of which were English language. The 100 most cited manuscripts for MICS are listed in Table 1 with a median citation number of 101 (51–414). The most cited article by Lichtenstein et al. [10] was published in Circulation and concentrated on transapical transcatheter aortic valve implantation in as a viable alternative to aortic valve replacement with cardiopulmonary bypass in selected patients with aortic stenosis. It was cited 414 times. The most recent study by Miceli et al. [47], looking at early outcomes and one-year survival following minimally-invasive aortic valve replacement with Perceval S sutureless valve in two European centres was published in the Journal of Thoracic and Cardiovascular Surgery and cited 63 times. The oldest featured manuscripts, of which there were four, were published in 1996 by Calafiore et al. investigating minimally-invasive coronary artery bypass grafting cited 110 times, Schwartz et al. exploring minimally-invasive cardiopulmonary bypass with cardioplegic arrest (a closed chest technique with equivalent myocardial protection) cited 106 times, Lytle’s examination of minimally-invasive cardiac surgery received 83 citations, and finally Stevens et al. with 68 citations for their examination of port-access coronary artery bypass with cardioplegic arrest.

**Table 1:** The 100 most cited articles in minimally-invasive cardiac surgery

Rank	Citations	First author	Rank	Citations	First author
*1*	414	Lichtenstein, SV [10]	*51*	74	Aris, A [11]
*2*	329	Kim, DH [12]	*52*	74	Sharony R
*3*	306	Cohn, LH [13]	*53*	72	Wierzbicki, M [14]
*4*	253	Pisano, GP [15]	*54*	72	Argenziano, M [16]
*5*	236	Armsby, LR [17]	*55*	71	DeRose, JJ [18]
*6*	232	Walther, T [19]	*56*	70	Savitt, MA [20]
*7*	223	Modi, P [21]	*57*	70	Dogan, S [22]
*8*	222	Mohr, FW [23]	*58*	70	Stephenson, ER [24]
*9*	201	Peyton, PJ [25]	*59*	68	Davis, Z [26]
*10*	189	Mohr, FW [27]	*60*	68	Treede, H [28]
*11*	167	Grossi, EA [29]	*61*	67	Stevens, JH [30]
*12*	162	Walther, T [31]	*62*	66	McGinn, JT [32]
*13*	160	Gundry, SR [33]	*63*	66	Seeburger, J [34]
*14*	154	Collura, CA [35]	*64*	65	Morgan, JA [36]
*15*	149	Diegeler, A [37]	*65*	65	Holzhey, DM [38]
*16*	146	Hu, P	*66*	65	Black, MD [39]
*17*	136	Schmitto, JD [40]	*67*	64	Subramanian, VA [41]
*18*	136	Walther, T [42]	*68*	64	Phan, K [43]
*19*	135	Degani, A [44]	*69*	63	Santarpino, G [45]
*20*	135	Grossi, EA [46]	*70*	63	Miceli, A [47]
*21*	132	Rosengart, TK [48]	*71*	63	Lang, N [49]
*22*	130	Nakamura, Y [50]	*72*	62	Roffi, M
*23*	128	Folliguet, TA [51]	*73*	61	Benetti, F [52]
*24*	128	Ye, J [53]	*74*	61	Van Linden, A [54]
*25*	126	Subramanian, VA [55]	*75*	61	Bonaros, N [56]
*26*	125	Chitwood, WR [57]	*76*	60	BhaskerRao, B [58]
*27*	116	Casselman, FP [59]	*77*	60	McClure, RS [60]
*28*	115	Dogan, S [61]	*78*	60	Holzhey, DM [62]
*29*	110	Calafiore, AM [63]	*79*	60	Kempfert, J [64]
*30*	108	Tabata, M [65]	*80*	59	Atallah, J [66]
*31*	106	Schwartz, DS [67]	*81*	59	Seeburger, J [68]
*32*	101	Kocher, AA [69]	*82*	58	Gillinov, AM [70]
*33*	100	Compton, FD [71]	*83*	58	Edgerton, JR [72]
*34*	92	Bacha, EA [73]	*84*	58	Plass, A [74]
*35*	87	Bein, B [75]	*85*	58	Kappert, U [76]
*36*	84	Glower, DD [77]	*86*	57	Calafiore, AM [78]
*37*	83	Buhre, G [79]	*87*	57	ElBardissi, AW [80]
*38*	83	Lytle, BW [81]	*88*	56	Gulielmos, V [82]
*39*	81	Holzhey, DM [83]	*89*	56	Dhole, S [84]
*40*	81	Dogan, S [85]	*90*	55	Wittwer, T [86]
*41*	80	Dogangil, G [87]	*91*	55	Iribarne, A [88]
*42*	80	Modi, P [89]	*92*	55	Formigari, R [90]
*43*	80	Stamou, SC [91]	*93*	54	Allen, KB [92]
*44*	80	Felger, JE [93]	*94*	54	Han, FT [94]
*45*	78	Galloway, AC [95]	*95*	54	Woo, YJ [96]
*46*	78	Holzhey, DM [97]	*96*	54	Navia, JL [98]
*47*	78	Reicher, B [99]	*97*	53	Reeves, BC [100]
*48*	77	Santana, O [101]	*98*	52	Bichell, DP [102]
*49*	76	Thiele, H [103]	*99*	51	Morgan, JA [104]
*50*	76	McVeigh, ER [105]	*100*	51	Sharony R [106]

Located after line 149

The 100 most cited manuscripts were from 30 different journals with between 1 and 35 articles per journal (Table 2). Most papers were published in the Annals of Thoracic Surgery (n = 35), also gaining the most citations (n = 3036). The New England Journal of Medicine had the highest impact factor (72.406) providing a single published article with 149 citations [37].

**Table 2:** List of journals from which the top 100 manuscripts are obtained

Journal title	Impact factor 2021	No. of manuscripts in top 100	No. of citations
NEW ENGLAND JOURNAL OF MEDICINE	91.245	1	149
NATURE MATERIALS	43.84	1	329
JOURNAL OF THE AMERICAN COLLEGE OF CARDIOLOGY	24.09	5	575
EUROPEAN HEART JOURNAL	29.98	1	63
SCIENCE TRANSLATIONAL MEDICINE	17.99	1	63
ANNALS OF SURGERY	12.97	1	306
BRITISH JOURNAL OF ANAESTHESIA	91.66	1	100
ANESTHESIOLOGY	7.067	1	201
HEART	5.994	1	56
CIRCULATION-ARRHYTHMIA AND ELECTROPHYSIOLOGY	6.568	1	54
HEART RHYTHM	6.343	1	154
JOURNAL OF THORACIC AND CARDIOVASCULAR SURGERY	5.209	14	1358
AMERICAN HEART JOURNAL	4.749	1	78
HEALTH TECHNOLOGY ASSESSMENT	4.058	1	54
54MEDICAL IMAGE ANALYSIS	8.545	1	74
ANESTHESIA AND ANALGESIA	5.178	1	87
MAGNETIC RESONANCE IN MEDICINE	4.668	1	76
SURGERY	3.356	1	54
EUROPEAN JOURNAL OF CARDIO-THORACIC SURGERY	4.191	10	1015
ANNALS OF THORACIC SURGERY	4.33	35	3036
AMERICAN JOURNAL OF PHYSIOLOGY-HEART AND CIRCULATORY PHYSIOLOGY	4.733	1	146
CIRCULATION	29.69	7	933
MANAGEMENT SCIENCE	4.219	1	253
CARDIOLOGY	1.791	1	61
JOURNAL OF CARDIOTHORACIC AND VASCULAR ANESTHESIA	1.58	2	139
JOURNAL OF CARDIAC SURGERY	1.351	3	172
PROCEEDINGS OF THE INSTITUTION OF MECHANICAL ENGINEERS PART H-JOURNAL OF ENGINEERING IN MEDICINE	1.617	1	80
JOURNAL OF HEART VALVE DISEASE	0.549	2	121

Located after line 155

The United States of America had the most publications (USA; n = 52; 5303 citations) followed by Germany (n = 27; 2598 citations) and Canada (n = 3; 681 citations). The United Kingdom had 3 manuscripts (244 citations) in the top 100. Harvard Medical School, Boston, Massachusetts, is the institution with the greatest number of papers in the top 100 (n = 6; 861 citations). A total of 9 first authors had more than one manuscript in the top 100 with one having 4 manuscripts (Holzhey, DM), while 2 others had 3 each (Dogan S; Walther, T) and 6 more each had 2 articles on the list.

The citation rate range of the top 10 papers was between 47 and 16.2 times (Table 3). The USA had the most papers in the top 10 with 4 manuscripts, Germany had 2 while Austria, Australia, Canada and France had 1 each.

**Table 3:** The 10 most cited Minimally-invasive Cardiac Surgery manuscripts

Rank	Citation rate	First author	Title	Country	Institution
1	47	Kim DH	Materials for multifunctional balloon catheters with capabilities in cardiac electrophysiological mapping and ablation therapy	USA	University of Illinois at Urbana Champaign, Illinois
2	34.5	Lichtenstein SV	Transapical transcatheter aortic valve implantation in humans—Initial clinical experience	Canada	St Paul's Hospital, University of British Columbia, Vancouver
3	25.8	Walther T	Transapical Aortic Valve Implantation: Step by Step	Germany	University of Leipzig, Leipzig
4	25.1	Peyton PJ	Minimally-invasive Measurement of Cardiac Output during Surgery and Critical Care A Meta-analysis of Accuracy and Precision	Australia	Austin Hospital, Melbourne
5	22.3	Modi P	Minimally-invasive mitral valve surgery: a systematic review and meta-analysis	USA	East Carolina Heart Institute, Greenville
6	21.3	Folliguet TA	Sutureless Perceval Aortic Valve Replacement: Results of Two European Centers	France	Institut Mutualiste Montsouris, Paris
7	20.2	Kocher AA	One-year outcomes of the Surgical Treatment of Aortic Stenosis With a Next Generation Surgical Aortic Valve (TRITON) trial: A prospective multicenter study of rapid-deployment aortic valve replacement with the EDWARDS INTUITY Valve System	Austria	Medical University of Vienna, Vienna
8	17.1	Collura CA	Left cardiac sympathetic denervation for the treatment of long QT syndrome and catecholaminergic polymorphic ventricular tachycardia using video-assisted thoracic surgery	USA	Mayo Clinic, Minnesota
9	16.7	Grossi EA	High-risk aortic valve replacement: Are the outcomes as bad as predicted?	USA	New York University School of Medicine, New York
10	16.2	Holzhey DM	Learning Minimally-invasive Mitral Valve Surgery A Cumulative Sum Sequential Probability Analysis of 3895 Operations From a Single High-Volume Center	Germany	Heart Centre Leipzig, Leipzig

Located after line 167

The top 100 manuscripts covered a wide range of subject areas. The number of papers relating to each topic is shown in Table 4. The most widely studied subjects were valve surgery with 42 manuscripts, followed by coronary revascularisation with 30 manuscripts. Both topics were explored together in 2 papers. Cardiac arrhythmias were the focus of 6 papers while the repair of septal defects and robotics each had 5.

**Table 4:** The number of manuscripts relating to each topic within MICS

Topic	Number
Valve surgery	42
Coronary revascularisation	30
Valve and revascularisation surgeries (combined)	2
Arrhythmias	6
ASD/VSD repair	5
Cardiac re-synchronization	2
Robotics	5
Cardiac output monitoring	2
Coronary artery fistulas	1
Training	1
Aortic procedures	1
Ministernotomy	1
Surgical glue	1

Located after line 174

**Fig. 1:**
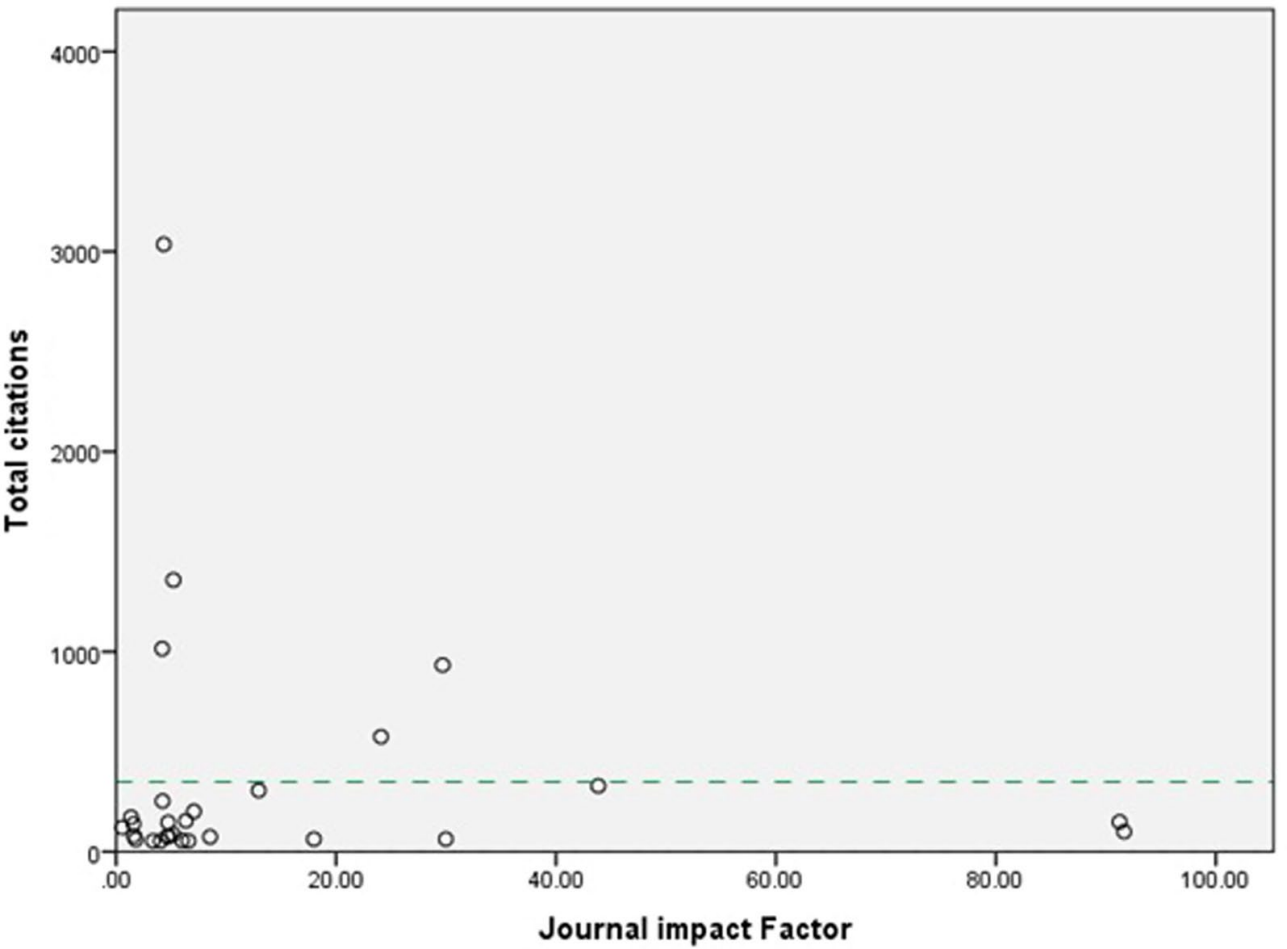
Relationship between journal impact factor and total citation. Regression analysis of impact factor and citation overall showed small positive correlation (R.^2^ = 0.006; *P* = 0.691)

**Fig. 2:**
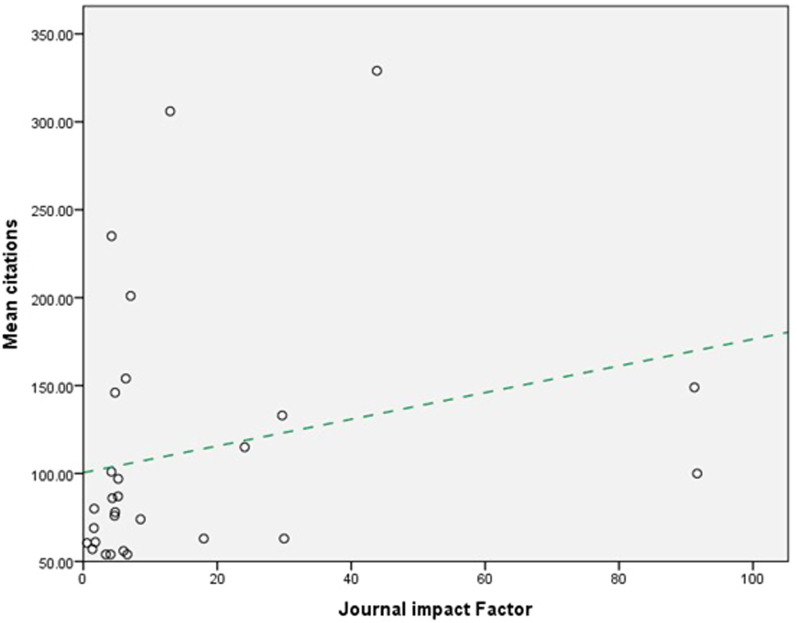
Relationship between journal impact factor and total citations. Mean citations were seen to rise with journal impact factor although not statistically significant, *P* = 0.208

Regression analysis of impact factor and citation overall showed small positive correlation (R^2^ = 0.006; *P* = 0.691) (Fig. 1). Mean citations were however seen to rise with journal impact factor (Fig. 2) (R^2^ = 0.6) this however was again shown to be a weak relationship (*P* = 0.208).

**Fig. 3:**
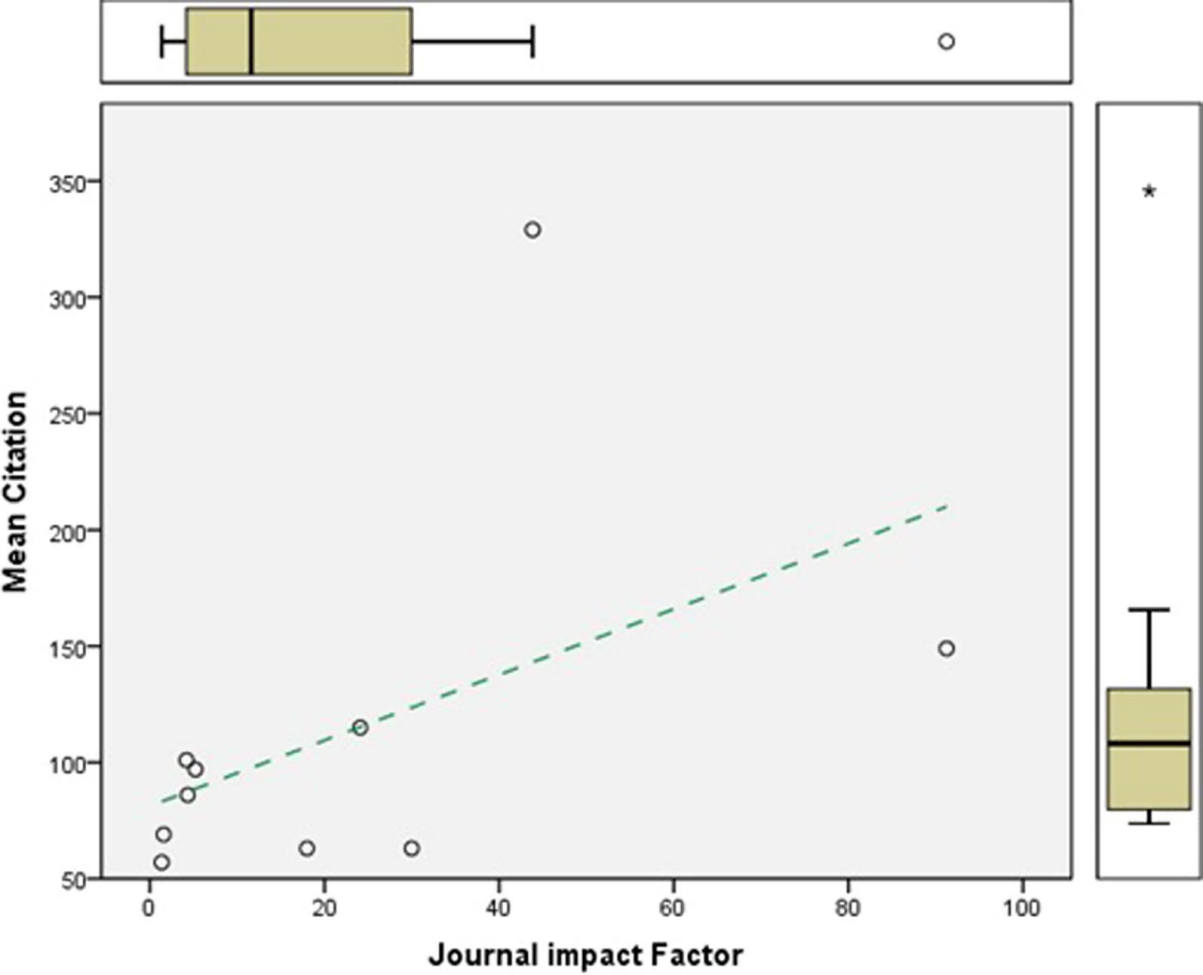
Relationship between journal impact fact and mean citations in cardiothoracic themed journals. A relationship appears to be present when comparing journal impact factor and citations an article receives (R^2^ = 0.239, *P* = 0.076) in cardiothoracic surgery themed journals

When focusing on purely cardiothoracic surgery specific journals, a more apparent relationship between journal impact factor and citations an article receives (R^2^ = 0.239, *P* = 0.076) (Fig. 3).

## Discussion

Being the first of its kind, this bibliometric analysis of MICS recognises the authors and topics possessing/holding the greatest effect/bearing within this surgical specialty. A number of pathological diseases are explored within the top 100 manuscripts, along with minimally-invasive surgical interventions to best manage these conditions.

Lichtenstein et al. [10] with 414 citations concentrated on transapical transcatheter aortic valve replacement in patients with severe symptomatic aortic stenosis and at unacceptably high risk for open aortic valve replacement with cardiopulmonary bypass due to comorbidity. Percutaneous transfemoral arterial valve implantation was unsuitable in these patients on account of iliofemoral atherosclerosis and, iliac and aortic tortuosity. Originally presented in an animal model by Andersen et al. [107] and first performed in humans as a transvenous transseptal procedure by Cribier et al. [108], several groups have followed the progression of percutaneous heart valves. A transfemoral arterial procedure has had promising outcomes though this approach is unsuitable in some patients due to femoral, iliac or aortic size, atheroma or tortuosity. It is concluded that prosthetic aortic valve implantation via transapical catheter-based approach without the need for cardiopulmonary bypass is an appropriate treatment option in patients that are unsuitable or represent an unacceptably high risk for either open or percutaneous procedures. The outcomes at 6-month follow-up [53] are also contained within the top 100, published in the European Journal of Cardio-thoracic Surgery and cited 128 times. Valve surgery is the dominant theme in the top 100 manuscripts, 8 of which focus on transapical aortic valve implantation.

The second most cited article by Kim et al. [12] (329 citations) published in Nature Materials Journal (impact factor 39.737) focused on the development of advanced minimally-invasive diagnostic and surgical tools, with examples given for complex arrhythmogenic cardiac conditions. They discuss the material challenges faced in finding biocompatible materials and devices that are of most value/advantageous in respect of the soft, curvilinear surfaces of the human body. Commercially available balloon catheters are exploited as a platform for such devices. Key steps in the construction process are presented to clarify how functionality is added to balloons without compromising their expansion or mechanical properties.

The third most cited article by Cohn et al. [13] (306 citations) published in the Annals of Surgery (impact factor 8.980) assessed the quality of valve replacement and repairs performed via minimally-invasive incisions compared with conventional open heart atrial and mitral valve surgery. Minimally-invasive surgery is shown to cause less trauma and blood loss, is cosmetically superior with less incisional pain and requirement for analgesia, and sternal infections are avoided. A disadvantage was identified in the use of femoral cannulation with this cohort experiencing groin infections and arterial reconstructions. A concern is highlighted regarding the quality of valve procedure achieved without complete exposure of the heart with results confirming equality between minimally-invasive and the traditional open technique. An additional advantage is emphasised for the cost-effective medical domain in which we reside.

In comparison to bibliometric analyses in other surgical specialties, the citation numbers of the top 100 manuscripts in MICS are significantly lower. For example, the most influential paper in the recent bibliometric analysis in minimally-invasive gastrointestinal surgery by Ahmad et al. [109] received 3331 citations with a median (range) citation of 555 (3331–317). The same is true in a number of other surgical and non-surgical specialties. This might indicate a low degree of research activity within MICS in comparison to more established fields. Alternatively, a lack of or limited funding may be responsible as evidence by only 6 of the top 100 citations being randomised trials, possibly due to the logistical challenges faced in undertaking such high quality clinical trials. It is more than likely, that as minimally-invasive techniques in cardiac surgery is still relatively new in the field, we are unable to recreate and follow these trends within the literature however, as this become more commonplace over time it is likely these tendencies will change and mirror those within other surgical specialities.

It was interesting to note that within this field of cardio surgery, there were ultimately poor relationships between citations accrued and journal impact factor. One thought would be that compared to specialities such as general surgery, cardiac surgery is still relatively in its infancy in its current form, this is further potentiated by the fact that MICS surgery is a very new subject area of interest. The weak relationships seen may be a factor of a lack of time in circulation and that this will strengthen as there are further development and time in the area.

The dominant themes identified in this analysis related to two subjects, valve surgery and coronary artery revascularisation, thus highlighting the areas in which most research activity is taking place.

A journal’s impact factor quantifies the average number of citations of a manuscript published within this journal over a given time period. Journals with a higher impact factor are considered as being of greater quality and have an increased likelihood of containing the most prominent publications.

Limitations of this study must be factored. Firstly, we only reviewed English language papers, this no doubt removes studies within this subject area that could limit our findings and further dilute any relation between journal impact factor and force. By focusing on only the top 100 papers based on citations, it must also be considered that other important research is left out, we aimed to account for this through also calculating citation rates however newer papers would still hold a disadvantage. There may also be an issue with citation rate in that older papers have more time to accumulate citations. This may create an evolving bias over time and thus citation rate itself may not be a true measure of influence on research. A further concern with bibliometric analysis would be that due to citations being affected by time, a papers influence is likely to change with new trends emerging as the scope of the field develops. As a result, newer publications will likely become more influential. This would mean a repeat analysis with the same methodoloy in 5 years will likely yield different findings with different topics and trends seen. The positive to this however is that such citation analyses will allow for quick analysis of the most impactful papers in a topic area at a time. This is highly pertinent in training whereby a trainee could identify papers with the most force to back up clinical decision making.

## Conclusions

Minimally-invasive cardiac surgery has gained popularity over the past decade, its growth propelled by the drive to welcome the benefits offered by minimal access techniques, for example reduced surgical trauma and less pain, to the realm of cardiac surgery. Indeed, patients seek surgical methods that allow for more rapid return to normal activities along with an improved quality of life.

Despite cardiac surgery’s progress towards less invasive techniques where the concomitant advances in perfusion methods, more sophisticated transthoracic echocardiography and innovations in robotic science are driving changes in clinical practice: Some doubts remain regarding its efficacy. The body of published literature on MICS has since grown greatly and there is a clear focus of development that is likely to impact future practice and training; with trainers and trainees needing to stay in the forefront of changes in surgical practice in Cardiac surgery.

This bibliometric analysis establishes an informative examination of the 100 most influential publications in MICS and outlines what constitutes a citable article. Undertaking a quantitative evaluation of the top 100 papers aids in recognising the contributions of key authors and institutions as well as guiding future efforts in this field to continually improve the quality of care offered to complex cardiac patients as well as providing focus for trainees and future researchers who can use such studies to identify and evaluate research that could later impact their clinical decisions and practice.

## Data Availability

The datasets generated and/or analysed during the current study are available in the Web of Science repository, https://clarivate.com/webofsciencegroup/solutions/web-of-science/.

## Abbreviations

MICS: Minimally-invasive cardiac surgery
MAVRIC: Manubrium-limited ministernotomy versus conventional sternotomy for aortic valve replacement
